# Standard Liver Volume-Predicting Formulae Derived From Normal Liver Volume in Children Under 18 Years of Age

**DOI:** 10.3389/fped.2021.629645

**Published:** 2021-02-19

**Authors:** Xintian Yang, Han Wang, Bingzi Dong, Bin Hu, Xiwei Hao, Xin Chen, Jing Zhao, Qian Dong, Chengzhan Zhu

**Affiliations:** ^1^Department of Pediatric Surgery, The Affiliated Hospital of Qingdao University, Qingdao, China; ^2^Medical College of Qingdao University, Qingdao University, Qingdao, China; ^3^Shandong Key Laboratory of Digital Medicine and Computer Assisted Surgery, The Affiliated Hospital of Qingdao University, Qingdao, China; ^4^Department of Radiology, The Affiliated Hospital of Qingdao University, Qingdao, China; ^5^Shandong College Collaborative Innovation Center of Digital Medicine Clinical Treatment and Nutrition Health, Qingdao University, Qingdao, China; ^6^Department of Hepatobiliary and Pancreatic Surgery, The Affiliated Hospital of Qingdao University, Qingdao, China

**Keywords:** liver volume, growth pattern, liver failure, hepatectomy, hepatoblastoma

## Abstract

**Background:** Standard liver volume (SLV) is important in risk assessment for major hepatectomy. We aimed to investigate the growth patterns of normal liver volume with age and body weight (BW) and summarize formulae for calculating SLV in children.

**Methods:** Overall, 792 Chinese children (<18 years of age) with normal liver were enrolled. Liver volumes were measured using computed tomography. Correlations between liver volume and BW, body height (BH), and body surface area (BSA) were analyzed. New SLV formulae were selected from different regression models; they were assessed by multicentral validations and were compared.

**Results:** The growth patterns of liver volume with age (1 day−18 years) and BW (2–78 kg) were summarized. The volume grows from a median of 139 ml (111.5–153.6 in newborn) to 1180.5 ml (1043–1303.1 at 16–18 years). Liver volume was significantly correlated with BW (*r* = 0.95, *P* < 0.001), BH (*r* = 0.92, *P* < 0.001), and BSA (*r* = 0.96, *P* < 0.001). The effect of sex on liver volume increases with BW, and BW of 20 kg was identified as the optimal cutoff value. The recommended SLV formulae were BW≤20 kg: *SLV* = *707.12* × *BSA*^1.09^; BW>20 kg, males: *SLV* = *691.90* × *BSA*^1.06^; females: *SLV* = *663.19* × *BSA*^1.04^.

**Conclusions:** We summarized the growth patterns of liver volume and provided formulae predicting SLV in Chinese children, which is useful in assessing the safety of major hepatectomies.

## Introduction

The most common liver tumor in children is hepatoblastoma (1). The main treatment of hepatoblastoma is surgical resection combined with neoadjuvant therapy (2). Extreme surgical resection after careful preoperative evaluation has similar overall survival rate with liver transplantation in some post-treatment extent (POST-TEXT) III and IV hepatoblastoma (3–5). It provides options for advanced-stage hepatoblastoma patients in the transplant waiting list and avoids long-term treatment of immunosuppression after transplantation (3, 6).

To perform liver resection successfully, the ratio of future remnant liver volume (FLV) to total normal liver volume is a crucial parameter in surgical planning (7, 8). A small FLV will lead to acute liver failure and even death in the perioperative period. In a previous study, preoperative total liver volume including tumors or preoperative measured total liver volume (mTLV, calculated by total liver volume–measured tumor volume) has been used to assess the FLV of patients (9). However, hepatoblastoma is generally large and normal liver tissue can be severely compressed by tumors. Therefore, the ratio of FLV to total liver volume including tumors will underestimate remnant liver volume. Meanwhile, FLV/mTLV is prone to overestimated future liver function, leading to post-hepatectomy liver dysfunction (9). In contrast, the standard liver volume (SLV) calculated by specific formulae indicates average hepatic volume level in people with normal livers. Some reports show that the FLV/SLV provides better estimates of postoperative liver function and has been well-applied in evaluating the risk of hepatectomy in adults (9, 10). However, there is no report about the normal liver volume and growth pattern of liver volume in children and only a few reliable reports on the SLV estimation in pediatric surgery (11–15).

We investigated growth patterns of liver volume in Chinese children, 1 day to 18 years old, and performed regression analysis to summarize the SLV formulae in pediatric patients.

## Patients and Methods

### Patients

Overall, 744 patients who underwent upper abdominal computed tomography (CT) between January 2013 and October 2019 at the Affiliated Hospital of Qingdao University were recruited as the local cohort. Additionally, 59 pediatric patients who underwent CT in 2019 at the Qingdao Women and Children's Hospital were recruited as the multicenter cohort. These children were treated in the hospital for appendicitis, ileus, intussusception, digestive tract foreign body, trauma, or other reasons. Premature infants among newborns were excluded. The sex, BW, and BH were recorded. Patients with hepatobiliary diseases that may affect the function or volume of the liver were excluded. Moreover, in the local cohort, we excluded one patient with diaphragmatic hernia and five with intestinal obstruction due to severe hepatic deformation or obvious effects of the intestinal contents on BW. Additionally, four cases of systemic inflammatory response syndrome were excluded because the livers were 1.3 times larger than the SLV during data validation. Finally, 733 cases (males, 434) were included in the study as the local cohort.

The local dataset was randomly divided into the training set (623 cases [85%]; age, 1 day−18 years, with median age of 6.5 years; males, 369, and females, 254) and local validation set (110 cases [15%]; age, 2 months−18 years, with median age of 7.1 years; males, 65, and females, 45). From the multicenter cohort, 59 patients (age, 1 month−14 years, with median age of 3 years; males, 31, and females, 28) were included as the multicenter validation set. Basic data included age (months), sex, BW (kg), BH (cm), and upper abdominal CT data. The Du Bois formula was used to calculate the body surface area (BSA; BSA = BW^0.425^×BH^0.725^×0.007184) (16).

### Liver Volumetry

A three-dimensional (3D) reconstruction software was widely used in the preoperative planning in liver surgery, including volumetry. We used CAS v2.2 (Hisense, Qingdao, China) to semi-automatically reconstruct stereo liver models from the upper abdominal CT Digital Imaging and Communications in Medicine (DICOM) files. Reconstruction procedures have been reported previously (17, 18). All the cases were rechecked by one experienced hepatobiliary surgeon and one radiologist by comparing the regions of interest on CT images with the corresponding zone of the 3D liver model. Every liver stereo model was rebuilt at least twice.

### Data Analysis and Regression

To explore if age, BW, BH, and BSA have linear correlations with the liver volume, we prepared the scatter plots. Subsequently, we analyzed the studentized residual error to ensure that the training set had a normal distribution. Stepwise regression was used to filter the variables that could be used as ideal predictors in multiple linear regression. Lastly, three different function models, namely, power function regression, multiple linear regression, and robust polynomial regression, were used to fit the training set.

### Validation of the Formulae

To determine whether the function models were suitable, we input the predictors of the training set into an artificial neuron network (ANN). ANN and other machine learning algorithms were implemented using PyTorch 1.2.0 (Facebook, Menlo Park, CA, USA). Local and multicenter sets were used to verify and assess the robustness and accuracy of the new formulae. To reduce bias and avoid circular arguments, the multicenter dataset was used in comparing the new formula and previous formulae.

## Results

### Growth Pattern of Liver Volume With Age and BW

**Figure 1:**
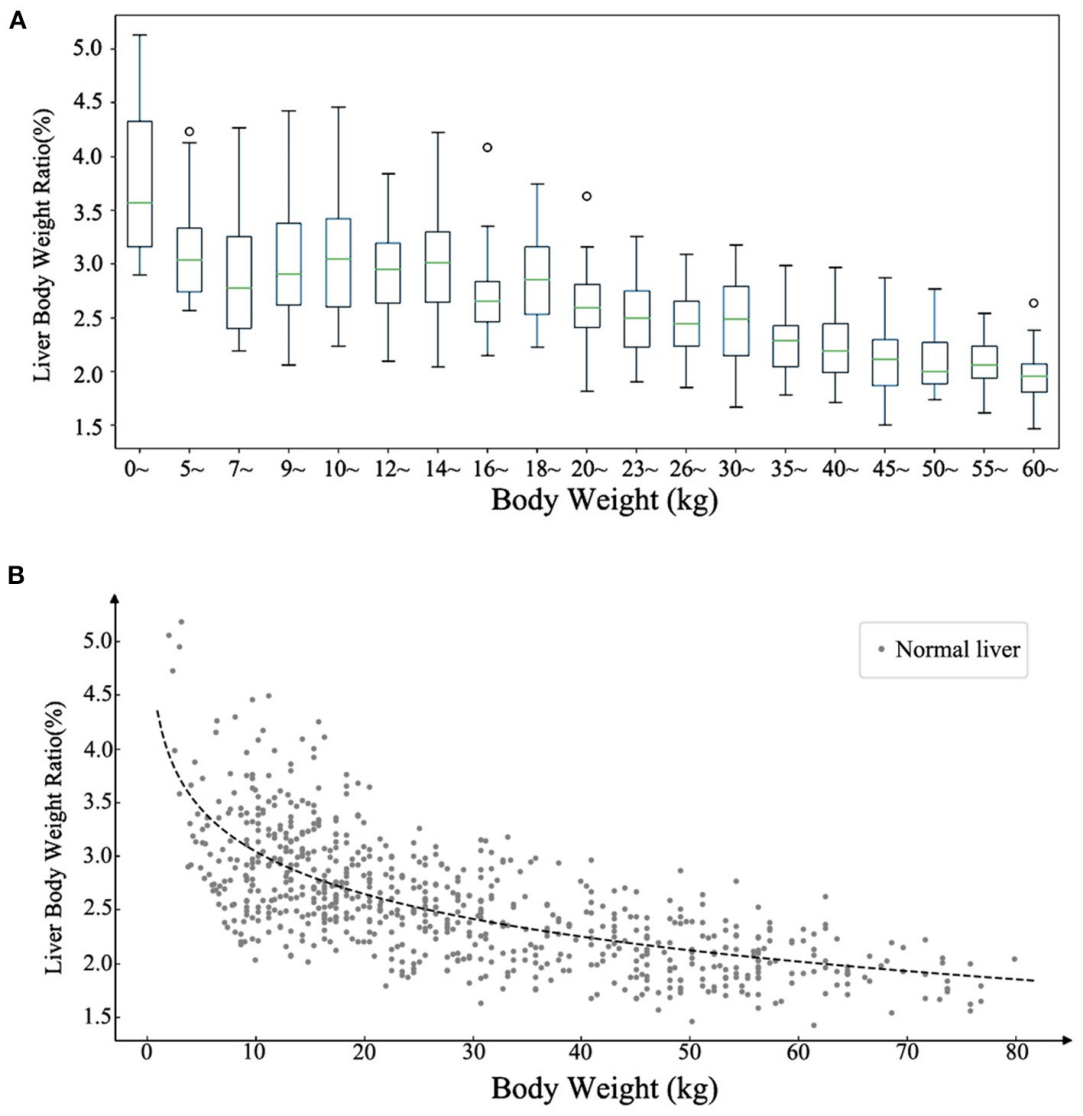
Distribution of the ratio of liver and body weights at different body weights. **(A)** The range of the normal ratio of liver and body weights with different body weights. **(B)** Scatter plot of the ratio of liver and body weights with body weight demonstrating a logarithmic descending trend.

All the cases of the local cohort were included in the statistical description of liver volume growth rule. The data characteristics of liver volumes at different ages are summarized in Table 1. The measured median liver volume varied from 139 ml (111.4–153.6 ml) in newborns to 1180.5 ml (1043–1303.1 ml) in those of 16–18 years of age. The range of liver body weight ratio (LBWR) at different BWs is illustrated in Figure 1A. LBWR revealed a logarithmic decreasing tendency with increase in BW (Figure 1B).

**Table 1:** Distribution of liver volume in children at different ages.

**Age^†^**	**Number of patients**	**Measured liver volume (ml)**	**Outliers**
0	8	139.0 (111.4–153.6)	0
1 m	13	156.3 (130.5–170.0)	0
2 m	17	184.1 (165.9–218.0)	2
4 m	15	196.9 (185.1–223.4)	1
6 m	13	250.0 (197.9–322.2)	0
8 m	12	283.2 (244.2–322.8)	0
10 m	12	274.0 (261.3–309.3)	0
12 m	39	301.3 (273.0–356.0)	0
18 m	17	367.8 (347.4–400.9)	0
24 m	36	391.8 (344.3–438.4)	1
30 m	19	406.4 (355.0–484.4)	0
3 y	43	426.9 (392.8–470.7)	0
4 y	52	511.3 (453.7–601.4)	1
5 y	43	533.5 (500.4–646.9)	0
6 y	44	600.9 (507.2–686.4)	0
7 y	36	659.5 (568.3–734.2)	1
8 y	43	735.0 (631.9–797.3)	3
9 y	42	733.9 (668.1–993.4)	0
10 y	31	848.2 (729.8–1072.0)	0
11 y	32	902.1 (739.6–1019.1)	0
12 y	48	1015.2 (923.2–1118.3)	3
13 y	58	1140.8 (942.8–1221.1)	0
14 y	20	1045.4 (860.8–1250.1)	0
16–18 y	40	1180.5 (1043–1303.1)	0

† *y, year; m, month*.

### New SLV Formulae and Validation

**Figure 2:**
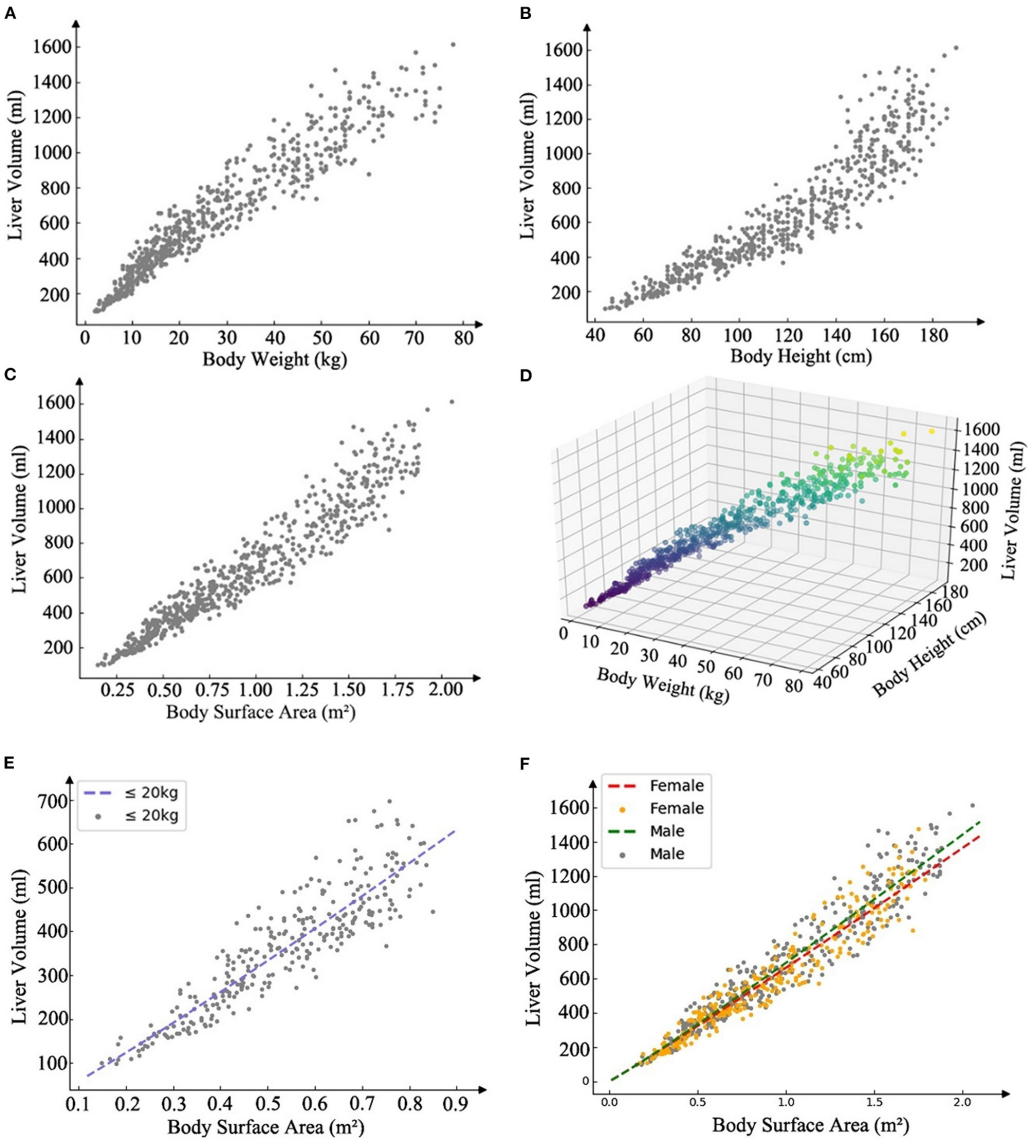
Scatter plot illustrating the linear correlation between the predictors and the liver volume, and present SLV formulae (power function) models and the scatter plot of the training set data. **(A)** Scatter plot of body weight and liver volume *r* = 0.95, *P* < 0.001. **(B)** Scatter plot of body height and liver volume *r* = 0.92, *P* < 0.001. **(C)** Scatter plot of body surface area and liver volume *r* =0.96, *P* < 0.001. **(D)** Scatter plot of body height and height and liver volume in a three-dimensional coordinate system. **(E)** Curve of SLV formula for weight <20 kg, *SLV* = *707.12* × *BSA*^1.09^. **(F)** Curve of SLV formula for weight more than 20 kg, males: *SLV*=*691.90*×*BSA*^1.06^ and females: *SLV*=*663.19*×*BSA*^1.04^*, BSA*=*BW*^0.425^ ×*BH*^0.725^×*0.007184* (DuBois formula).

Liver volume was significantly correlated with age (*r* = 0.90; *P* < 0.001), BW (*r* = 0.95; *P* < 0.001), BH (*r* = 0.92, *P* < 0.001), and BSA (*r* = 0.96, *P* < 0.001) (Figure 2). The residual error of the training set was normal. BSA was used in power function regression (univariate regression). After stepwise regression, BH, BW, and sex were used in multiple linear regression function (*P* < 0.001). The differences in SLV between the sexes were insignificant when the children were young; however, the differences increased as the children grew older. According to previous reports and tests of cutoff values at different BWs, the formulae for children under 20 kg were listed separately. Therefore, we generated three formulae: one for BW<20 kg and two for males and females with BW>20 kg.

**Figure 3:**
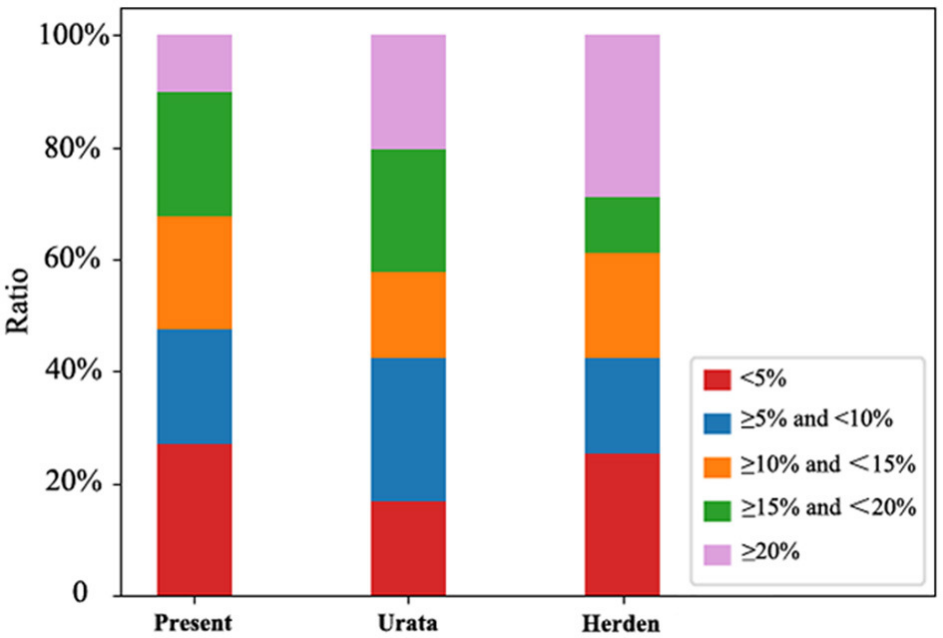
Distribution of predicted value in different intervals of MAPE. The distribution of the predicted values of Urata, Herden and the present formulae in different MAPE intervals.

The local and multicenter validation results of different models are summarized in Table 2. The multicenter validation results revealed a mean absolute percentage error (MAPE) of 11.5%, which proved that the regression models we used were acceptable. The model based on power function with the lowest MAPE in two validations (Figure 2) was the most accurate across the three models. The following new formulae for SLV (ml) based on Chinese children under 18 years of age (Table 3) were derived from BSA (BW≤20 kg, *SLV*=*707.12*×*BSA*^1.09^ (*P* < 0.001); BW>20 kg for males, *SLV*=*691.90*×*BSA*^1.06^, and for females, *SLV*=*663.19*×*BSA*^1.04^) and the Du Bois BSA formula (*BSA*=*BW*^0.425^×*BH*^0.725^×*0.007184*). In these formulae, BW is measured in kg and BH in centimeters. Compared with previously reported formulae, the new SLV formulae were significantly more accurate in estimating the liver volume in the multicenter dataset (MAPE, 11.6%; root of mean square error [RMSE], 72.8) (Figure 3). According to the result of multicenter validation, poor predictive performance in young children and newborn of Urata formula causes larger MAPE and RMSE. Herden formulae were derived from Caucasian children, and its predicted values at all ages were significantly larger than the actual liver volume.

**Table 2:** Formulae of different methods of regression and validation results based on the local validation and multicenter validation sets.

**Dataset**	**Method**	Regression function^*^	***r*^2^**	**Local validation**		**Multicenter validation**	
				**MAPE%**	**RMSE**	**MAPE%**	**RMSE**
Entire training set	Power function	Male: *SLV = 691.90×BSA^1.06^*^†^	0.94	10.5	91.48	10.9	79.37
		Female: *SLV = 663.19×BSA^1.04^*	0.94	11.0	90.30	10.8	55.27
	Multiple linear	Male: *SLV=2.94×BH+12.95×BW-50.01*	0.93	10.5	94.41	11.7	80.25
		Female: *SLV=2.53×BH+13.07×BW-30.31*	0.92	11.7	94.41	11.5	59.72
	Robust polynomial	Male: *SLV=3.08×BH+12.99BW-72.68*	0.93	10.6	94.46	11.3	82.81
		Female: *SLV=2.48×BH+13×BW-27.65*	0.93	11.6	93.99	11.4	59.41
≤20 kg training set	Robust polynomial	SLV=*3.02×BH+13.32×BW-70.06*	0.82	10.1	54.01	11.3	51.98
	Power function	SLV=*707.12×BSA^1.09^*	0.84	10.2	53.09	10.8	50.87

* *P < 0.001*.

† *Du Bois formula: BSA = BW^0.425^ ×BH^0.725^×0.007184; SLV, standard liver volume (ml); BSA, body surface area (m^2^); BH, body height (cm); BW, body weight (kg)*.

**Table 3:** Recommended formulae that use power function and robust polynomial regression.

	Formula^*^
Power function regression	Male: *SLV = 691.90×BSA^1.06^*^†^
	Female: *SLV = 663.19×BSA^1.04^*
	Under 20 kg: *SLV=707.12×BSA^1.09^*
Robust polynomial regression	Male: *SLV=3.08×BH+12.99BW-72.68*
	Female: *SLV=2.48×BH+13×BW-27.65*
	Under 20 kg: *SLV=3.02×BH+13.3×BW-70.06*

* *P < 0.001*;

† *Du Bois formula: BSA = BW^0.425^ ×BH^0.725^×0.007184; SLV, standard liver volume (ml); BSA, body surface area (m^2^); BH, body height (cm); BW, body weight (kg)*.

## Discussion

The FLV/SLV is an important factor evaluating risk of major hepatectomy (8, 11, 19–21). There have been several SLV formulae for adult (12, 13, 22, 23). However, pediatric SLV formulae are rare. In this study, we summarized the normal liver volumes of 792 Chinese children and calculated their correlation with body weight and body height. By regression analysis, we proposed new formulae for estimating SLV in children and validated them in an external cohort.

Urata formula, the most widely used SLV formula in liver surgery of adults, only involved 65 Japanese children (11), making it difficult to avoid overfitting of the model. The predicted volume in young children usually has a large error (15). Herden formulae were derived from the data of exsanguinous livers of 388 Caucasian children following autopsies (14), in which the exsanguinous livers may slightly vary from alive livers. Meanwhile, the differing nationalities may make Herden formulae not applicable to oriental children. In order to estimate the risk for extreme hepatectomy more accurately, further investigations of new SLV formulae need to be performed based on a larger pediatric population.

Compared with the previous formulae (11, 14), larger dataset was involved and age and sex differences were included in the SLV formulae in this study. We utilized multicenter data to verify the reliability of the new formulae, which compensated for the lack of validation of previous studies, and a different evaluation indicator, MAPE—which is better applicable to pediatric data—was used to evaluate the predictive effects of the SLV formulae. We introduced the ANN algorithm for the first time to assess if regression models of the SLV formulae were suitable. Furthermore, we systematically summarized the ranges of liver volume and LBWR at different ages or BW in children. Additionally, we firstly summarized the normal liver volume in Chinese children under 18 years old and demonstrated the growth pattern of the normal liver volume with age and BW. Table 1 and Figure 1 demonstrate the ranges of liver volume and LBWR at different ages and BWs in healthy children, indicating the individual differences and distributions. The reason for eliminating age and choosing BW as a parameter of LBWR was to observe the rule that growth retardation is common in children with end-stage liver diseases, and BW can reflect the nutritional status of children better. These big data research may provide basis for future research about growth and nutrition of children.

Calculating SLV is more complicated in children than in adults due to changes in BW and BH owing to overall growth. We separately listed a formula for children <20 kg of BW due to the following reasons. First, based on the experience of previous formulae, the variance in liver volume and absolute errors in older children is larger than that in smaller infants. Thus, the weights of data from older children exceed those of young infants in regression, leading to poor fitting of the formula in young infants. Second, differences in SLV due to sex were insignificant in younger children but increased as they grew up. Third, we tried 10 kg, 15 kg, and some other values as the cutoff values; however, we found that 20 kg was the ideal cutoff to allow the formulae to fit the training set. Therefore, we listed one formula for children with BW≤20 kg and one each for males and females with BW>20 kg. According to the validation results, the difference between power function and robust polynomial function was extremely small, and the polynomial function is recommended to facilitate calculations (Table 3).

In children, as the liver volume increases with age and BW, the absolute error of predicted value increases as well, which could not reflect the fitting effects of the formulae in the training sets with patients of different ages. Additionally, overfitting is probable in small datasets due to the size and quality of the training set, which can result in false better *r*^2^ and lower the accuracy of the models. Furthermore, *r*^2^ can be an objective parameter to assess the accuracies of different SLV formulae in children only if the training sets are comparable. *r*^2^ of SLV formulae containing children's data reportedly were significantly bigger than that of formulae that used only adults' data. However, the accuracies of their predictions are not always consistent with the corresponding *r*^2^, which is probably due to the different ages included in the training sets (11–13, 22). To avoid the common pitfalls of SLV studies, MAPE was chosen in evaluating the accuracy of the formulae. Additionally, ANN can fit linear models and can match non-linear models; therefore, we introduced this machine learning algorithm to SLV studies for the first time to ensure the fitting effects of the function models. Furthermore, to reduce bias and avoid circular arguments, a multicenter dataset was used to compare the new and old formulae (11, 14).

From the clinical aspect, According to previous studies, over 0.3 FLV/SLV was acceptable in major hepatectomy in adults (7). Confronted with advanced-stage liver tumors in children, surgeons can use the present formulae to evaluate whether postoperative liver volume is enough to make it possible for surgeons to perform extreme hepatectomies in advanced-stage liver tumors of children and avoid liver transplantation and long-term immunosuppressive therapy (10).

There were some limitations to this study. We only collected data of Chinese children, and nationality differences should be considered when applying the new formulae. Comparisons with the Herden formula proved that the liver volume in Caucasian children is larger than that in their Asian peers (14, 15). Additionally, owing to the insufficient number of clinical cases at our center, more prospective clinical studies are required to validate the findings of the current study.

In conclusion, we firstly summarized the growth pattern of liver volume with age and BW and reported new formulae for predicting SLV of Chinese children 0–18 years of age. Combined with 3D reconstruction, the new formulae are useful in assessing the risk of major hepatectomy.

## Data Availability Statement

The original contributions generated for the study are included in the article/supplementary material, further inquiries can be directed to the corresponding authors.

## Ethics Statement

The studies involving human participants were reviewed and approved by The Ethics Committee of the Affiliated Hospital of Qingdao University. Written informed consent to participate in this study was provided by the participants' legal guardian/next of kin.

## Author Contributions

XY, CZ, and QD: conception and design. CZ and QD: administrative support. XY, BH, XH, and JZ: provision of study materials or patients. XY, HW, and BD: collection and assembly of data. XY, HW, BD, BH, XH, XC, CZ, and QD: data analysis and interpretation. All authors: manuscript writing and final approval of manuscript.

## Conflict of Interest

The authors declare that the research was conducted in the absence of any commercial or financial relationships that could be construed as a potential conflict of interest.
